# Association Between Insulin Resistance Indices and Liver Function Parameters Among Women With Polycystic Ovary Syndrome

**DOI:** 10.1002/edm2.490

**Published:** 2024-05-20

**Authors:** Marzieh Saei Ghare Naz, Maryam Mousavi, Faezeh Firouzi, Amir Abbas Momenan, Fereidoun Azizi, Fahimeh Ramezani Tehrani

**Affiliations:** ^1^ Reproductive Endocrinology Research Center Research Institute for Endocrine Sciences, Shahid Beheshti University of Medical Sciences Tehran Iran; ^2^ Department of Pathology, School of Medicine Shahid Beheshti University of Medical Sciences Tehran Iran; ^3^ Prevention of Metabolic Disorders Research Center Research Institute for Endocrine Sciences, Shahid Beheshti University of Medical Sciences Tehran Iran; ^4^ Endocrine Research Center Research Institute for Endocrine Sciences, Shahid Beheshti University of Medical Sciences Tehran Iran; ^5^ The Foundation for Research & Education Excellence Vestaria Hills AI USA

**Keywords:** insulin resistance, liver function tests, metabolic syndrome, polycystic ovary syndrome

## Abstract

**Objective:**

This study aimed to investigate whether polycystic ovary syndrome (PCOS) status changes the association between insulin resistance (IR) indices and liver function parameters among women.

**Methods:**

This is a cross‐sectional, population‐based study. We selected 1101 subjects aged ≥20 years from participants of Tehran Lipid and Glucose Study (TLGS). All of them had known the status of PCOS, and all variables were related to the IR indices and liver function parameters. The main outcome measures were TG/HDL‐C and triglyceride‐glucose (TyG) and liver function parameters (hepatic steatosis index [HSI], alanine transaminase [ALT] and aspartate transaminase [AST]).

**Result:**

In the present study, there was no significant difference between the PCOS and the non‐PCOS regarding the presence of liver function abnormalities. A model adjusted by age and BMI showed that the upper tertile of TyG index was positively associated with high AST (OR = 3.04 [95% CI: 1.20–7.68], *p* < 0.05), high ALT (4.76 [3.07–7.36], *p* < 0.05) and high HSI (8.44 [1.82–39.17], *p* < 0.05). Although the history of diabetes had a positive impact on elevated AST (1.66 [1.15, 2.40], *p* < 0.05), the third tertile of TG/HDL‐C was associated with increased odds of elevated ALT (3.35 [2.21–5.06]) and HSI (6.55 [1.17–36.46]), whereas the second tertile of TG/HDL‐C (OR = 2.65, CI 95%: 1.74–4.03) was also positively associated with elevated ALT. PCOS had no significant association with elevated liver function tests.

**Conclusion:**

The highest tertile of TyG index and the TG/HDL‐C ratio as a surrogate of IR might play a role in detecting abnormalities of liver function parameters among women. However, PCOS status cannot change the association between IR and liver dysfunction.

## Graphical abstract

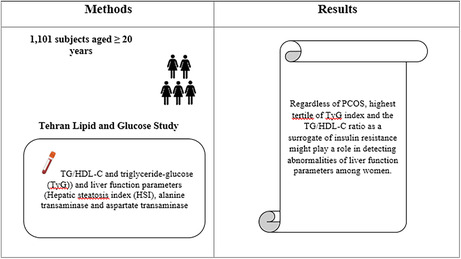

## 1 Introduction

Insulin resistance (IR) is known as an impaired biological response to stimulation of insulin in target tissues [1]. IR is most likely to contribute to metabolic abnormalities [2]. Along with increasing metabolic syndrome (MET) worldwide, IR as a dominant factor of METs has also seen a rising tide [3, 4]. In people with IR, failing to inhibit hepatic glucose production and increasing liver lipid synthesis can result in hypertriglyceridaemia [5]. IR is also strongly associated with liver disease such as a nonalcoholic fatty liver disease (NAFLD) [6, 7]. Today, a number of surrogate indexes have been identified as IR markers. The triglycerides/high‐density lipoprotein cholesterol (TG/HDL‐C) ratio and the triglyceride‐glucose (TyG) index are considered ideal substitutional IR markers [8, 9, 10]. Furthermore, TyG index and TG/HDL‐C are independently associated with liver function parameters [11, 12].

Meanwhile, polycystic ovary syndrome (PCOS) is a significant reproductive disorder and endocrinopathy affecting women worldwide [13, 14, 15]. The prevalence rate of PCOS according to different diagnostic criteria among Iranian women was estimated between 13 and 19% [16]. Evidence suggested the various reproductive, cutaneous, metabolic and psychological sequelae [17, 18]. PCOS is also linked to obesity, altered glucose metabolism, pregnancy complications, cardiovascular diseases (CVDs) and gynaecological malignancies [19]. Androgen excess is a key feature of PCOS, and it is suggested that it plays a predominant role in metabolic perturbations in women with PCOS [20]. The risk of METs in women with PCOS is almost 3.5 times higher than that in those without PCOS [21], and more than half of women with PCOS suffer from IR [22, 23]. Recently, evidence highlighted the hepatic feature of METs in women with PCOS. Women with PCOS have an increased risk of elevated liver enzymes and NAFLD [24, 25]. Liver function tests including alanine transaminase (ALT) and aspartate transaminase (AST) are the markers of hepatocellular injury [26]. Previous studies have also revealed that the concentrations of AST and ALT are associated with risk of Type 2 diabetes mellitus (T2DM) [27, 28, 29]. Recently, Targher et al. demonstrated that in women with PCOS with normal levels of ALT, insulin sensitivity was similar to the control group. In contrast, it was significantly lower among women with elevated ALT levels [30]. Furthermore, they found that in PCOS women with elevated ALT, there was an abnormality in lipid profile [30].

A recent meta‐analysis has shown that the prevalence of NAFLD is higher among women with PCOS [31]. Despite compelling evidence suggesting that NAFLD is a feature of PCOS, the association between liver function parameters and insulin markers has not been clearly described. Hence, because of a lack of adequate population‐based evidence, and considering the steep rise of metabolic disease around the world, this study aimed to investigate whether PCOS status changes the association between IR indices and liver function parameters among the population of the Tehran Lipid and Glucose Study (TLGS).

## 2 Method

### 2.1 Study Design

The present cross‐sectional study was conducted in the context of the TLGS, a prospective, population‐based cohort study, which aimed to determine the risk factors for noncommunicable diseases among a representative Tehran urban population. This study was initiated in 1999 and has six subsequent follow‐up visits, approximately every 3 years. In‐person interviews were conducted by qualified professionals at the TLGS unit; they also performed physical and clinical examinations. The interview included questions on obstetrics and reproductive history, with a focus on the regularity of the menstrual cycle, hyperandrogenic symptoms, parity and adverse events related to the pregnancy. Clinical assessment included general anthropometrics and physical examinations including hirsutism using the modified Ferriman–Gallwey scoring method. Further details regarding rationale and design of the ongoing TLGS have been reported previously [32, 33]. This study was approved by the Ethical Review Board of the Research Institute for Endocrine Science. All participants signed informed consent forms, which adhered to the Declaration of Helsinki's basic principles, and the study was also approved by the Research Institute for Endocrine Sciences' ethics committees.

### 2.2 Study Population

For the present study, of women participating in the sixth follow‐up visit, we selected a total of 1101 women 20 years and older with recorded PCOS status, and the recorded information of AST, ALT and hepatic steatosis index (HSI) was included. All women with no recordation of PCOS status and women under 20 years were excluded. As 13‐ to 19‐year‐old females are considered adolescent and the aim of this study was limited to adults, they were excluded.

### 2.3 Measurements

Venous blood was drawn from all study individuals between 7:00 and 9:00 AM, 12–14 h after they had stopped eating and 2–3 h after waking up. On the day of blood collection, all blood lipid analyses were carried out in the TLGS research facility using the Selectra 2 autoanalyser (Vital Scientific, Spankeren, the Netherlands). Using glycerol phosphate oxidase, cholesterol esterase and cholesterol oxidase, respectively, serum TG and total cholesterol were determined using enzymatic calorimetric techniques. After precipitating the phosphotungstic acid–carrying lipoproteins containing apolipoprotein B, HDL‐C levels were measured. Total cholesterol and HDL‐C had intra‐ and inter‐assay CVs of 0.5% and 2%, respectively; TG had CVs of 0.6% and 1.6%, respectively. The standard colorimetric Jaffe kinetic reaction method was used to assess the levels of serum creatinine, with sensitivity set at 0.2 mg/dL and inter‐ and intra‐assay CVs of 2.5% and 1.9%, respectively. Serum liver enzymes were assayed using enzymatic colorimetric methods. Weight and height measurements were performed using a digital scale with an accuracy of 100 g, and a stadiometer with an accuracy of nearest 0.5 cm was used for measuring height.

### 2.4 Definition of Terms

PCOS was defined according to the 2003 Rotterdam ESHRE/ASRM‐sponsored PCOS consensus workshop criteria 1 as endorsed by the most recent international evidence‐based guideline [34]. Elevated liver enzymes are considered as AST >35 IU/L and ALT >25 IU/L [35, 36]. Hepatic steatosis index was defined as (HSI) = 8 × (ALT/AST) + BMI + (2, if diabetes mellitus) + (2, if female), with values <30 ruling out and values >36 ruling in steatosis [37]. IR markers were defined as TyG = Ln [TG (mg/dL) × FBS (mg/dL)/2] [38], and TG/HDL ratio was calculated as TG (mg/dL)/HDL‐cholesterol (mg/dL).

Smoking status was categorised as never‐smoker and current‐smoker. Physical activity was assessed using the Modifiable Activity Questionnaire [27] and defined as low and high levels, with METs < 600 min‐week‐1 considered as low physical activity. T2DM was defined as fasting blood sugar (FBS) ≥126 mg/dL or 2‐h postload glucose ≥200 mg/dL or taking medication for diagnosed diabetes [39]. Hypertension (HTN) was defined as systolic blood pressure (SBP) ≥140 mmHg, diastolic blood pressure (DBP) ≥90 mmHg or using antihypertensive medications. Dyslipidaemia was defined as hypertriglyceridaemia (TG ≥150 mg/dL), hypo‐HDL (HDL <40 mg/dL) and/or using lipid‐lowering drugs [40]. A set of heart and blood vessel problems is referred to as CVD. In this study, chronic kidney disease (CKD) was considered an estimated glomerular filtration rate below 60 mL/min/1.73 m [41].

### 2.5 Statistical Analysis

The baseline characteristics of participants were described and compared on the basis of tertile categories of TyG index and TG/HDL‐C ratio. The analysis of variance and Kruskal–Wallis tests were used for continuous variables with or without normal distribution, respectively. The chi‐squared test or Fisher's exact test was also used for categorical variables. TyG index and TG/HDL‐C ratio were fitted as categorical variables (stratified into three subgroups: T1<8.53; 8.53≤T2<9; 9≤T3 for TyG and T1<2.04; 2.04≤T2<3.33; 3.33≤T3 for TG/HDL). The lowest baseline TyG index and TG/HDL‐C ratio categories were used as a reference to compute the odds ratios (ORs) and 95% confidence intervals (CIs).

Logistic regression was used to identify the baseline prognostic factors that potentially predicted each elevated enzyme status as the dependent binary variables (ALT<25 and ≥25, AST<35 and ≥35, HSI⪡30 and ≥30). In models, the confounding variables with collinearity, tested by a calculated variance inflation factor (VIF) ≥10, were removed. Thus, modified Model 1 adjusted by age and BMI. Model 2 further adjusted by age, BMI, dyslipidaemia, physical activity, smoking, T2DM, CVD, HTN, CKD status and PCOS group. Pearson correlation test was used to analyse the association between IR indices and liver function parameters. All confounders were selected according to the literature review and available data.

Data analysis was conducted on R version 4.1.1 and IBM SPSS Statistics version 21 (IBM Corp., Armonk, NY, USA). All statistically significant results were reported at *p* < 0.05.

## 3 Results

A total of 1101 women participated in this study. Tables 1 and 2 present the characteristics of the study population according to tertiles of the TyG index and TG/HDL‐C ratio. As shown, there were significant differences in terms of median BMI among PCOS and non‐PCOS women in tertiles of TyG index and TG/HDL‐C. Moreover, frequency of T2DM was significantly different among PCOS and non‐PCOS women in tertiles of TG/HDL index. There were no significant differences between elevated and normal levels of liver function parameters in tertiles of TyG index and TG/HDL‐C ratio.

**TABLE 1:** Characteristics of the participants according to tertiles of TyG index.

Variables	Tertiles of TyG index								
	Tertile 1 (*n* = 358)		*p*	Tertile 2 (*n* = 362)		*p*	Tertile 3 (*n* = 381)		*p*
	PCOS (*n* = 82)	Non‐PCOS (*n* = 276)		PCOS (*n* = 84)	Non‐PCOS (*n* = 278)		PCOS (*n* = 77)	Non‐PCOS (*n* = 304)	
Age (years), median (IQR)	50 (43.75–55)	51 (45–59)	0.009	49.80 (6.40)	55.18 (8.26)	<0.001	52.88 (6.07)	57.51 (7.92)	<0.001
BMI (kg/m^2^), median (IQR)	28.19 (25.46–31.79)	27.33 (24.77–30.22)	0.12	29.26 (4.29)	29.75 (4.80)	0.40	31.02 (4.59)	30.45 (4.88)	0.35
Smoker									
Yes	3 (3.7%)	7 (2.5%)	0.70	3 (3.6%)	20 (7.2%)	0.23	3 (3.9%)	10 (3.3%)	0.73
No	79 (96.3%)	269 (97.5%)		81 (96.4%)	258 (92.8%)		74 (96.1%)	294 (96.7%)	
Physical activity									
Low	51 (62.2%)	197 (71.4%)	0.11	59 (70.2%)	176 (63.3%)	0.24	56 (72.7%)	231 (76.0%)	0.55
High	31 (37.8%)	79 (28.6%)		25 (29.8%)	102 (36.7%)		21 (27.3%)	73 (24.0%)	
Dyslipidaemia									
Yes	38 (46.3%)	130 (47.1%)	0.90	53 (63.1%)	192 (69.1%)	0.30	75 (97.4%)	292 (96.1%)	0.74
No	44 (53.7%)	146 (52.9%)		31 (36.9%)	86 (30.9%)		2 (2.6%)	12 (3.9%)	
T2DM									
Yes	6 (7.3%)	29 (10.5%)	0.39	13 (15.5%)	37 (13.3%)	0.61	29 (37.7%)	130 (42.8%)	0.41
No	76 (92.7%)	247 (89.5%)		71 (84.5%)	241 (86.7%)		48 (62.3%)	174 (57.2%)	
CVD									
Yes	0 (0.0%)	6 (2.2%)	0.34	2 (2.4%)	12 (4.3%)	0.53	1 (1.3%)	19 (6.2%)	0.09
No	82 (100.0%)	270 (97.8%)		82 (97.6%)	266 (95.7%)		76 (98.7%)	285 (93.8%)	
HTN									
Yes	7 (8.5%)	43 (15.6%)	0.10	18 (21.4%)	73 (26.3%)	0.37	22 (28.6%)	111 (36.5%)	0.19
No	75 (91.5%)	233 (84.4%)		66 (78.6%)	205 (73.7%)		55 (71.4%)	193 (63.5%)	
CKD									
Yes	31 (37.8%)	92 (33.3%)	0.45	27 (32.1%)	130 (46.8%)	0.01	39 (50.6%)	177 (58.2%)	0.23
No	51 (62.2%)	184 (66.7%)		57 (67.9%)	148 (53.2%)		38 (49.4%)	127 (41.8%)	
AST									
Normal	82 (100.0%)	270 (97.8%)	0.34	80 (95.2%)	268 (96.4%)	0.74	72 (93.5%)	284 (93.4%)	0.97
Elevated	0 (0.0%)	6 (2.2%)		4 (4.8%)	10 (3.6%)		5 (6.5%)	20 (6.6%)	
ALT									
Normal	75 (91.5%)	251 (90.9%)	0.88	65 (77.4%)	215 (77.3%)	0.99	48 (62.3%)	206 (67.8%)	0.36
Elevated	7 (8.5%)	25 (9.1%)		19 (22.6%)	63 (22.7%)		29 (37.7%)	98 (32.2%)	
HSI									
Normal	4 (4.9%)	24 (8.7%)	0.25	2 (2.4%)	3 (1.1%)	0.32	1 (1.3%)	2 (0.7%)	0.49
Elevated	78 (95.1%)	252 (91.3%)		82 (97.6%)	275 (98.9%)		76 (98.7%)	302 (99.3%)	

Abbreviations: ALT, alanine transaminase; AST, aspartate transaminase; BMI, body mass index; CKD, chronic kidney disease; CVD, cardiovascular disease; FBS, fasting blood sugar; HIS, hepatic steatosis index; HTN, hypertension; PCOS, polycystic ovary syndrome; T2DM, type 2 diabetes mellitus; TLGS, Tehran Lipid and Glucose Study; TyG, triglyceride‐glucose.

**TABLE 2:** Characteristics of the participants according to TG/HDL‐C ratio index.

Variables	Tertiles of TG to HDL index								
	Tertile 1 (*n* = 360)		*p*	Tertile 2 (*n* = 372)		*p*	Tertile 3 (*n* = 369)		*p*
	COS (*n* = 76)	Non‐COS (*n* = 284)		COS (*n* = 82)	Non‐COS (*n* = 290)		COS (*n* = 85)	Non‐COS (*n* = 284)	
Age (years), median (IQR)	49.58 (7.73)	53.61 (8.51)	<0.001	51.41 (6.84)	55.64 (8.30)	<0.001	50.92 (5.97)	56.12 (8.37)	<0.001
BMI (kg/m^2^), median (IQR)	28.19 (25.03–32.00)	27.43 (24.88–30.35)	0.30	29.46 (4.64)	29.98 (5.19)	0.38	30.85 (27.12–33.64)	29.71 (27.20–33.61)	0.24
Smoker									
Yes	2 (2.6%)	10 (3.5%)	1.00	4 (4.9%)	16 (5.5%)	0.82	3 (3.5%)	11 (3.9%)	0.88
No	74 (97.4%)	274 (96.5%)		78 (95.1%)	274 (94.5%)		82 (96.5%)	273 (96.1%)	
Physical activity									
Low	46 (60.5%)	201 (70.8%)	0.08	58 (70.7%)	189 (65.2%)	0.34	62 (72.9%)	214 (75.4%)	0.65
High	30 (39.5%)	83 (29.2%)		24 (29.3%)	101 (34.8%)		23 (27.1%)	70 (24.6%)	
Dyslipidaemia									
Yes	23 (30.3%)	112 (39.4%)	0.14	58 (70.7%)	218 (75.2%)	0.41	85 (100.0%)	284 (100.0%)	—
No	53 (69.7%)	172)60.6%(		24 (29.3%)	72 (24.8%)		—	—	
T2DM									
Yes	47 (16.5%)	3 (3.9%)	0.005	22 (26.8%)	67 (23.1%)	0.48	23 (27.1%)	82 (28.9%)	0.74
No	237 (83.5%)	73 (96.1%)		60 (73.2%)	223 (76.9%)		62 (72.9%)	202 (71.1%)	
CVD									
Yes	0 (0.0%)	7 (2.5%)	0.35	1 (1.2%)	11 (3.8%)	0.47	2 (2.4%)	19 (6.7%)	0.18
No	76 (100.0%)	277 (97.5%)		81 (98.8%)	279 (96.2%)		83 (97.6%)	265 (93.3%)	
HTN									
Yes	6 (7.9%)	57 (20.1%)	0.01	19 (23.2%)	73 (25.2%)	0.71	22 (25.9%)	97 (34.2%)	0.15
No	70 (92.1%)	227 (79.9%)		63 (76.8%)	217 (74.8%)		63 (74.1%)	187 (65.8%)	
CKD									
Yes	29 (38.2%)	110 (38.7%)	0.92	34 (41.5%)	133 (45.9%)	0.48	34 (40.0%)	156 (54.9%)	0.01
No	47 (61.8%)	174 (61.3%)		48 (58.5%)	157 (54.1%)		51 (60.0%)	128 (45.1%)	
AST									
Normal	76 (100.0%)	275 (96.8%)	0.21	78 (95.1%)	275 (94.8%)	1.00	80 (94.1%)	272 (95.8%)	0.55
Elevated	0 (0.0%)	9 (3.2%)		4 (4.9%)	15 (5.2%)		12 (4.2%)	5 (5.9%)	
ALT									
Normal	69 (90.8%)	254 (89.4%)	0.73	61 (74.4%)	217 (74.8%)	0.93	58 (68.2%)	201 (70.8%)	0.65
Elevated	7 (9.2%)	30 (10.6%)		21 (25.6%)	73 (25.2%)		27 (31.8%)	83 (29.2%)	
HSI									
Normal	5 (6.6%)	22 (7.7%)	0.73	1 (1.2%)	6 (2.1%)	1.00	1 (1.2%)	1 (0.4%)	0.40
Elevated	262 (92.3%)	71 (93.4%)		81 (98.8%)	284 (97.9%)		84 (98.8%)	283 (99.6%)	

Abbreviations: ALT, alanine transaminase; AST, aspartate transaminase; BMI, body mass index; CKD, chronic kidney disease; CVD, cardiovascular disease; DBP, diastolic blood pressure; HIS, hepatic steatosis index; HTN, hypertension; PCOS, polycystic ovary syndrome; SBP, systolic blood pressure; T2DM, type 2 diabetes mellitus; TLGS, Tehran Lipid and Glucose Study; TyG, triglyceride‐glucose.

Regarding the prevalence of abnormal liver function tests, by increasing the tertiles of TyG index and TG/HDL‐C, the prevalence of high ALT and AST, and HSI was increased (Tables 1 and 2).

Table 3 shows multivariable‐adjusted logistic regression for abnormal liver function tests in tertiles of indices insulin markers. There was no significant difference in the model, except for high ALT. A model adjusted by age and BMI showed that the upper tertile of TyG index was positively associated with high AST (OR = 3.04 [95% CI: 1.20–7.68], *p* < 0.05), high ALT (4.76 [3.07–7.36], *p* < 0.05) and high HSI (8.44 [1.82–39.17], *p* < 0.05). Furthermore, BMI was positively associated with elevated ALT, ALT and HSI. PCOS had no significant association with elevated liver function tests, whereas a history of T2DM had a positive impact on elevated AST (1.66 [1.15, 2.40], *p* < 0.05; Table 3).

**TABLE 3:** Multivariable‐adjusted logistic regression for abnormal liver function tests in tertiles of indices insulin markers.

	High AST		High ALT		High HSI	
	Model 1	Model 2	Model 1	Model 2	Model 1	Model 2
TyG index						
Tertile 1	1	1	1	1	1	1
Tertile 2	1.86 (0.69–4.99)	1.93 (0.71–5.27)	**2.80 (1.80–4.37)**	**2.64 (2.64–4.16)**	2.88 (0.87–9.49)	1.86 (0.50–6.84)
Tertile 3	**3.04 (1.20–7.68)**	2.81 (1.001–7.93)	**4.76 (3.07–7.36)**	**3.85 (2.37–6.23)**	**8.44 (1.82–39.17)**	2.29 (0.37–14.19)
Age	1.02 (0.98–1.06)	1.01 (0.97–1.06)	0.99 (0.97–1.01)	0.98 (0.96–1.003)	1.003 (0.95–1.05)	0.98 (0.92–1.05)
BMI	**1.08 (1.02–1.14)**	**1.07 (1.01–1.14)**	**1.04 (1.009–1.07)**	**1.03 (1.002–1.06)**	**2.94 (2.17–3.99)**	**3.16 (2.24–4.47)**
Dyslipidaemia (ref = no)	—	0.89 (0.36–2.18)	—	1.13 (0.74–1.71)	—	1.80 (0.58–5.58)
Physical activity (ref = low)	—	0.92 (0.45–1.89)	—	1.14 (0.82–1.59)	—	2.40 (0.78–7.37)
Smoking (ref = no)	—	0.46 (0.06–3.57)	—	1.36 (0.68–2.72)	—	1.68 (0.12–23.20)
T2DM (ref = no)	—	1.54 (0.76–3.09)	—	**1.73 (1.21–2.47)**	—	1**
CVD (ref = no)	—	1.33 (0.36–4.89)	—	1.24 (0.60–2.58)	—	0.07 (0.0003–18.29)
HTN (ref = no)	—	1.10 (0.55–2.23)	—	1.19 (0.83–1.71)	—	1.16 (0.22–6.10)
CKD (ref = no)	—	0.92 (0.47–1.79)	—	1.02 (0.74–1.41)	—	2.93 (0.92–9.28)
PCOS (ref = no)	—	1.009 (0.68–1.49)	—	1.02 (0.85–1.23)	—	0.95 (0.50–1.83)
TG/HDL						
Tertile 1	1	1	1	1	1	1
Tertile 2	1.80 (0.76–4.27)	1.71 (0.69–4.21)	**2.65 (1.74–4.03)**	**2.47 (1.58–3.86)**	1.87 (0.66–5.25)	0.94 (0.94–3.45)
Tertile 3	1.70 (0.71–4.05)	1.46 (0.54–3.93)	**3.35 (2.21–5.06)**	**2.99 (1.84–4.86)**	**6.55 (1.17–36.46)**	2.97 (0.37–23.70)
Age	1.02 (0.98–1.06)	1.01 (0.97–1.06)	1.002 (0.98–1.02)	0.98 (0.96–1.008)	1.01 (0.96–1.07)	0.98 (0.92–1.05)
BMI	**1.08 (1.02–1.14)**	**1.07 (1.01–1.14)**	1.04 (1.01–1.07)	**1.03 (1.002–1.06)**	**2.81 (2.11–3.74)**	**3.25 (2.27–4.64)**
Dyslipidaemia (ref = no)	—	1.07 (0.43–2.68)	—	1.03 (0.66–1.60)	—	1.88 (0.55–6.44)
Physical activity (ref = low)	—	0.91 (0.44–1.85)	—	1.13 (0.81–1.57)	—	2.65 (0.86–8.09)
Smoking	—	0.45 (0.05–3.47)	—	1.36 (0.68–2.71)	—	1.46 (0.10–19.68)
T2DM (ref = no)	—	1.78 (0.91–3.50)	—	**2.03 (1.43–2.87)**	—	1**
CVD (ref = no)	—	1.32 (0.36–4.84)	—	1.18 (0.56–2.44)	—	0.08 (0.0005–12.80)
HTN (ref = no)	—	1.14 (0.56–2.31)	—	1.24 (0.87–1.78)	—	1.19 (0.22–6.22)
CKD (ref = no)	—	0.99 (0.51–1.92)	—	1.09 (0.79–1.49)	—	3.01 (0.95–9.53)
PCOS (ref = no)		1.0006 (0.67–1.48)	—	1.01 (0.84–1.21)	—	0.93 (0.48–1.77)

*Note:* Data are expressed as odds ratios (ORs) and 95% confidence intervals (CIs) in the parenthesis. Model 1: adjusted by age and BMI. Model 2: adjusted by Model 1 + dyslipidaemia, physical activity, smoking, T2DM, CVD, HTN, CKD status and PCOS group.

Abbreviations: BMI, body mass index; CVD, cardiovascular disease; CKD, chronic kidney disease; HTN, hypertension.

* For the highlighted values *p* < 0.05.

** Because of the lack of variation in frequency of T2DM between two groups, the odds ratio cannot be estimated.

When it comes to the TG/HDL‐C ratio, in Model 1, the third tertile of TG/HDL‐C was associated with increased odds of elevated ALT (3.35 [2.21–5.06]) and HSI (6.55 [1.17–36.46]). The second tertile of TG/HDL‐C was also positively associated with elevated ALT 2.65 (1.74–4.03). Being PCOS had no significant association with elevated liver function tests.

**FIGURE 1:**
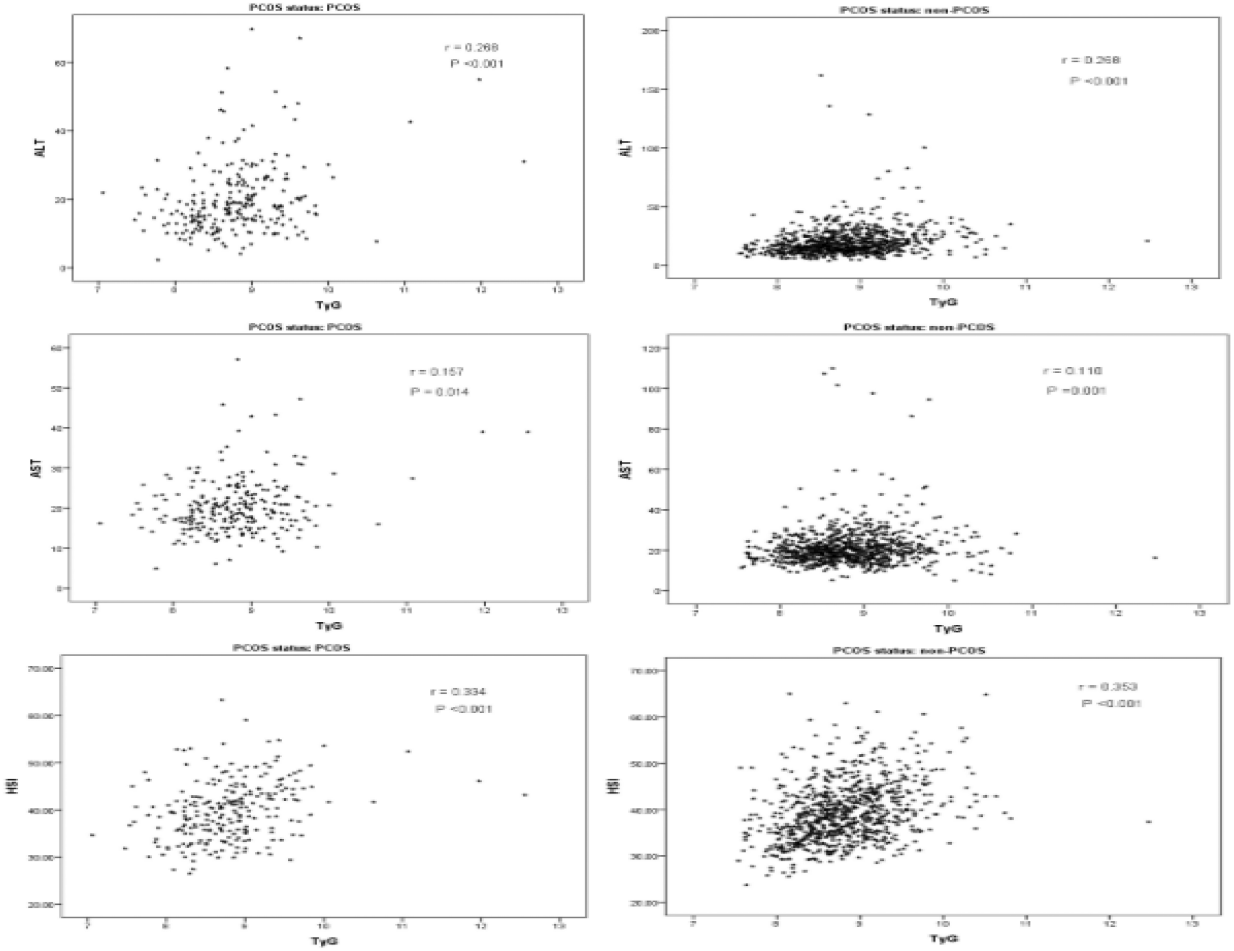
Correlation analysis of the TyG with liver function parameters.

**FIGURE 2:**
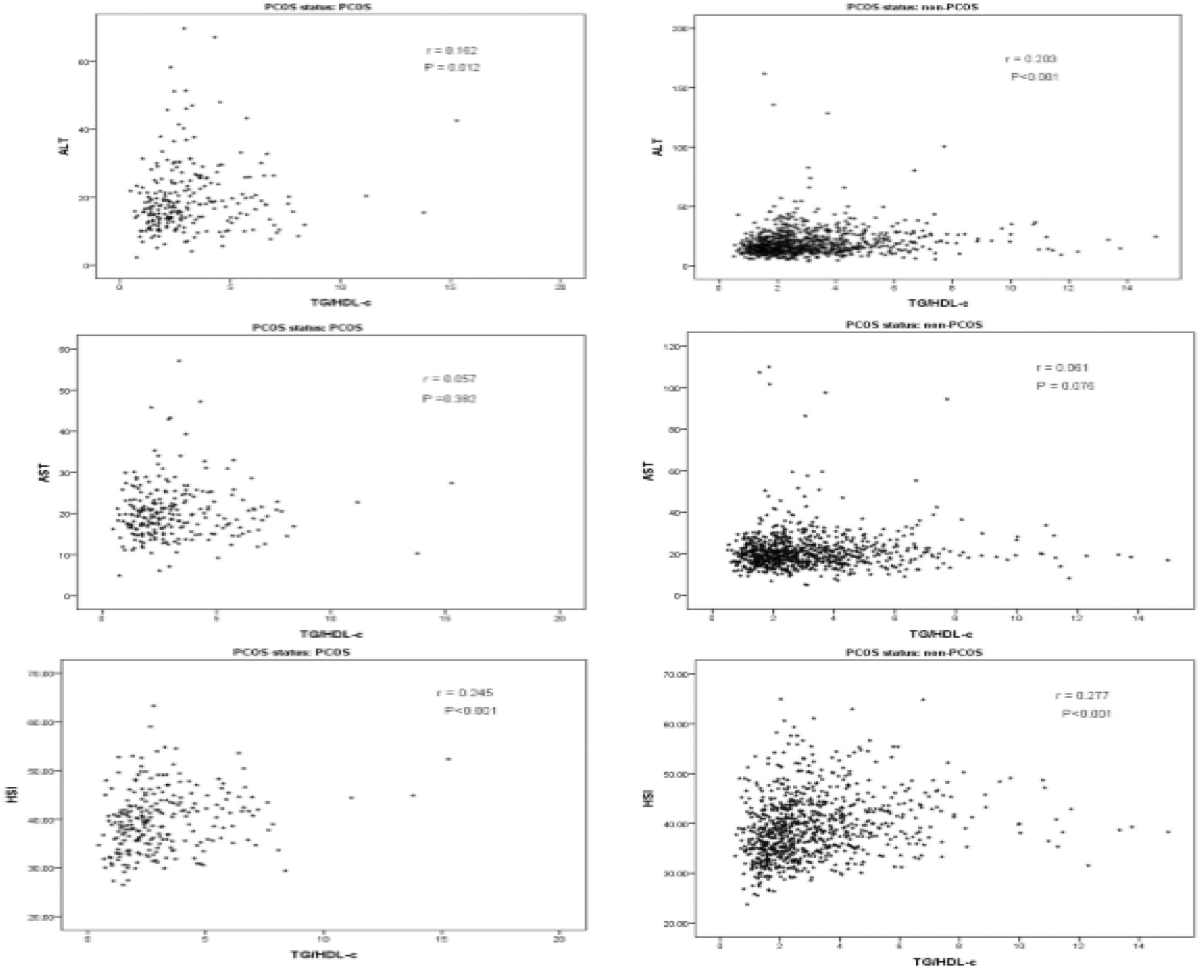
Correlation analysis of the TG/HDL with liver function parameters.

Figure 1 shows that TyG was significantly correlated with ALT levels, AST levels and HSI among both women with and without PCOS. Furthermore, TG/HDL‐C was significantly correlated with ALT levels, and HSI among women with PCOS and without PCOS (Figure 2).

## 4 Discussion

This study examined the associations of IR surrogate markers on the risk of abnormalities in liver function parameters among Iranian women with and without PCOS. The results showed that the risk of elevated liver enzyme and HSI increased across tertiles of the TyG index and TG/HDL‐C, and PCOS had no significant association with odds of elevated liver function tests. Compared with the lowest tertile of the TyG index, the highest tertile (third tertile) was associated with a greater odds of the elevated AST, ALT and HSI. The highest tertile of TyG index and the TG/HDL‐C ratio were found to be useful tools in detecting abnormalities of liver function parameters. BMI and a history of T2DM were also positively associated with abnormal liver function after adjusting for potential confounders. Furthermore, the correlation analysis showed that IR indices correlated positively with liver function parameters in women with PCOS. Further research is needed to better understand the relationship between PCOS and liver disease. Liver disease accounts both direct costs and substantial disease burden worldwide [42]. NAFLD is the common cause of asymptomatic elevated liver enzymes [43]. According to a recent meta‐analysis, the prevalence of NAFLD in women was about 30% [44]. In a study among the Iranian population, the risk of fatty liver diseases increased among subjects with IR [45]. As the liver disease developed silently and because of delay in diagnosis, prognosis is poor, it is worthwhile to start widespread screening for liver disease [46, 47, 48]. Liver biochemical tests are commonly used to assess liver function, and elevated liver enzymes can serve as early warning signs for liver and metabolic disease [49, 50, 51]. This is because liver is a core organ in which lipid and glucose metabolism regulation occur [52]. It is also well‐believed that IR is a key factor in the pathophysiology of liver disease, and lipid markers such as the TG/HDL‐C ratio and TyG index are useful tools for evaluating metabolic dysregulations like insulin levels and IR in women with PCOS [53, 54, 55, 56, 57].

In our study, the PCOS status did not change the risk of elevated ALT, AST or HSI. To our knowledge, there is no other study with a similar objective for comparison with our findings. Elevated liver enzymes are a common feature among women with PCOS [58]. Furthermore, dyslipidaemia is also seen more frequently among women with PCOS [59]. Liu et al. found that fasting insulin is considered a mediator between PCOS and NFALD [60]. In a study among Korean women with PCOS, lipid profile of participants was in the normal range and insulin level in 75 g glucose tolerance test (GTT) and BMI>25 kg/m^2^ were not significantly associated with subsequent NAFLD [61]. It is possible because of the higher similarity between the PCOS and non‐PCOS groups, and the association was not significant between two groups.

Our finding showed the strong association between T2DM and increased the risk of elevated levels of ALT after adjustment for potential confounders. After adjusting for confounders, the highest quartile of TyG index and TG/HDL‐C ratio were significantly associated with an increased risk of elevated AST, ALT and HSI. These results support recent evidence suggesting a link between IR and liver dysfunction. Previous studies have shown that IR in women with PCOS can have detrimental effects on liver function [62]. The TyG index has been identified as a practical tool for screening the risk of NAFLD [63]. The study by Liu et al. demonstrated an association between IR, obesity and elevated liver enzymes [64]. Previous research has also shown a link between IR and liver function parameters. A study of Taiwanese adults found that those in the highest quintile of the TyG index had a higher risk of elevated AST, ALT and ALP than those in the median quintile [65]. A recent study of Chinese individuals found that IR was significantly associated with an increased risk of elevated ALT, AST and y‐glutamyl transferase (GGT) [64]. Additionally, serum ALT concentrations have been identified as an independent predictor of diabetes in another study [66]. The conflicting results of previous studies may be due to differences in participant characteristics, variable definitions and measurements. Several credible mechanisms have been proposed underlying the association between IR and disturbed liver function. The mechanisms underlying this association include inflammation, alterations in lipid metabolism, lipotoxicity, oxidative stress and cytokine‐induced liver injury [67, 68].

The main clinical implication of this study is that clinicians can consider TyG index and the TG/HDL‐C ratio as a surrogate of IR for detection of liver function abnormalities among women.

Our study has some limitations and strengths. The population‐based setting of study lets us have the representative sample of PCOS population because of reducing selection bias. In addition, adjustment of the related potential confounders produced valuable results. However, the cross‐sectional design of this study prevents us from making causal inferences. Furthermore, the lack of data regarding liver biopsy or ultrasonography makes it impossible to identify NAFLD. Additionally, lifestyle modifications that could provide more precise information for tracking liver disease in PCOS were not assessed in this study. Furthermore, as some abnormalities are temporarily and we have no data on duration of abnormality of liver tests, it is suggested future studies consider it. Prospective population‐based studies with long enough follow‐up and comprehensive assessments including several liver function tests and ultrasonography assessments are recommended for a better understanding of the effect of PCOS on liver dysfunction.

## 5 Conclusion

This population‐based study showed the highest tertile of TyG index and the TG/HDL‐C ratio as a surrogate of IR might play a role in detecting abnormalities of liver function parameters among women. However, PCOS status cannot change the association between IR and liver dysfunction. The focus of assessment of liver function of women should be broadened to the surrogate IR markers, which are easily accessible.

## Author Contributions

All authors conceived the study, participated in its design and helped to draft the manuscript. Likewise, all authors made suggestions and critical reviews to the initial draft and contributed to its improvement until reaching the final manuscript, which was read and approved by all authors.

## Ethics Statement

The study was approved by the Ethics Committee of the Research Institute for Endocrine Sciences, Shahid Beheshti University of Medical Sciences (IR.SBMU.ENDOCRINE.REC.1401.026). Informed consent was obtained in accordance with the Declaration of Helsinki. All methods were carried out in accordance with relevant guidelines and regulations with the Declaration of Helsinki.

## Consent

The authors have nothing to report.

## Conflicts of Interest

All authors declare no conflict of interest.

## Data Availability

The datasets used and/or analysed during the current study are available from the corresponding author upon reasonable request.
